# Mechanisms for community prevention of violence against women in low- and middle-income countries: a realist approach to a comparative analysis of qualitative data

**DOI:** 10.1016/j.socscimed.2022.115064

**Published:** 2022-05-25

**Authors:** Hattie Lowe, Laura Brown, Ayesha Ahmad, Nayreen Daruwalla, Lu Gram, David Osrin, Krishna Panchal, Daniella Watson, Cathy Zimmerman, Jenevieve Mannell

**Affiliations:** a Institute for Global Health, University College London; b St George’s, University of London; c SNEHA (Society for Nutrition, Education and Health Action), Mumbai; d University of Southampton; e London School of Hygiene and Tropical Medicine

**Keywords:** Violence against women, community-based interventions, intervention development research, India, Afghanistan, Peru, Rwanda

## Abstract

Growing evidence suggests that community-based interventions in low- and middle-income countries (LMICs) can effectively address harmful social norms that promote or sustain gender inequality and drive violence against women (VAW). However, understanding what actions communities are already taking to address harmful social norms and prevent VAW is an essential first step for intervention development. Towards this goal, drawing on collective action theory, we conducted a realist analysis of secondary qualitative data collected with communities in India, Afghanistan, Peru and Rwanda. We coded interview and focus-group data from 232 participants to identify the contexts, mechanisms and outcomes (CMO) relevant for community action. We synthesized CMO configurations from each dataset into a conceptual framework composed of three middle-range theories of mechanisms driving community action to prevent VAW in LMICs. Our results highlight the importance of dedicated spaces for discussing VAW, VAW leaders as positive role models, and community perceptions of VAW as a problem worthy of intervention. In Rwanda and Peru, there was strong evidence to support the operation of these mechanisms. Contextual factors, including national and local policy and programmes targeting VAW, activated mechanisms that led to community action. In India and Afghanistan, evidence for the presence of these mechanisms was weaker, with social norms about women’s position and violence being a private family matter preventing communities from addressing violence. Despite contextual differences, our data demonstrated communities in all four settings were somewhere along a pathway of change towards VAW prevention. This supports the need to build future prevention interventions on pre-existing mechanisms that trigger community action, rather than implementing existing interventions without local adaptation. Our conceptual framework serves as a tool for assessing these mechanisms of community action as part of intervention development research, centring community knowledge and fostering local ownership for more relevant and sustainable VAW prevention interventions.

## Introduction

Intimate partner violence (IPV), the most common form of violence against women (VAW), affects one in three women globally, with severe consequences for physical and mental health (WHO, 2021). While many VAW prevention interventions target individual and relationship behaviours, growing evidence suggests community-based interventions in low- and middle-income countries (LMICs) can effectively address the harmful social norms that promote or sustain gender inequality and drive VAW (Ellsberg *et al.,* 2015; Heise and Kotsadam, 2015; Jewkes, Stern and Ramsoomar, 2019). For example, *SASA!*, a community mobilisation trial in Uganda, reported positive effects on emotional IPV experience among women in intervention communities (Abramsky *et al.,* 2016). Other participatory group-based training interventions such as *Yaari Dosti* with men and boys in India (Verma, Pulerwitz and Magendra, 2008) and *Stepping Stones* with men and women in South Africa (Gibbs *et al.,* 2020) have also demonstrated significant reductions in IPV perpetration by men.

The focus on community-based interventions is a welcome shift away from individual behaviour change interventions for VAW prevention, such as perpetrator rehabilitation programmes (Wilson, Feder and Olaghere, 2021) and women’s self-defence and empowerment initiatives (Hollander, 2014; Jordan and Mossman, 2019). Interventions targeting perpetrators often use cognitive behavioural therapy approaches, which rely inherently on men changing their own behaviour rather than changing the broader social structures and ideals of masculinity in which men are situated (Jewkes, Flood and Lang, 2015). Similarly, women’s self-defence or empowerment initiatives have been critiqued for blaming the victim, not addressing underlying gender norms that perpetuate violence (Hollander, 2016) and failing to address the need to build collective agency (Gram, Morrison and Skordis-Worrall, 2019).

In contrast to individualised programming, community-focused VAW prevention interventions aim to disrupt the gendered social norms that condone VAW and support male dominance (Heise and Kotsadam, 2015). Social norms refer to collectively held beliefs about what others believe or do (Cialdini, Kallgren and Reno, 1991), and VAW researchers draw on this concept to consider how shared beliefs about gender roles and the pervasiveness of violence can, for example, influence an individual’s own use of violence (Orchowski, 2019). VAW prevention interventions in LMIC settings have used several techniques to disrupt social norms of violence, including community activism (Abramsky *et al.,* 2016), group training (Gibbs *et al.,* 2020), and participatory decision-making (Shannon and Mannell, 2021). In high-income countries, a substantial body of work has focused on the potential for bystander interventions to prevent sexual violence in higher education settings (Banyard, Moynihan and Crossman, 2009), drawing on evidence that men’s perceptions of the social norms around violence are associated with their own violent behaviours and their likelihood of intervening in cases of violence (Loh *et al.,* 2005).

Social psychologists interested in community mobilisation approach social norms from a different perspective, arguing that a reduction in violence comes about through challenging symbolic contexts in which it is accepted and normalised (Mannell and Dadswell, 2017). Community activists are seen as putting pressure on the more powerful actors who control the symbolic context through campaigning, court action, acts of civil disobedience, and public protest (Campbell *et al.,* 2010). This highlights the potential for intervention researchers to not only think about how they might challenge individual perceptions of what others in a community believe about violence (social norms), but to also consider the role that collective action by communities can play in preventing violence through symbolically challenging its acceptance and use.

While theoretically compelling, social norms may be limited in identifying the range of actions communities can take towards violence prevention. Empirical research from multiple settings highlights the diversity of actions community members use to prevent violence, including making noise when violence occurs (Singhal, Wang and Rogers, 2013; Colvin and Karcher, 2020), establishing neighbourhood watch groups, offering group-based emotional support for women’s stories to be heard (Nagar, 2000; Wagman *et al.,* 2015), and imposing restrictions on alcohol use (Wilson, Graham and Taft, 2014). These actions that communities may already be taking are not usually captured by research that focuses exclusively on social norms as beliefs about the expectations of others and the influence this can have on individual behaviours.

As a means of developing conceptual understandings of VAW prevention at the community level, we present a realist analysis (Pawson and Tilley, 1997) of mechanisms instigating community action to prevent VAW, using empirical evidence from India, Afghanistan, Peru and Rwanda. This complements the VAW prevention literature on social norms by identifying additional community-based mechanisms that may lead to VAW prevention, with the aim of expanding current thinking on how to prevent VAW in partnership with communities.

### Conceptualising community actions to address VAW

We draw on social theories of collective action to move beyond a focus on social norms theory in community-based VAW prevention research. Collective action, a theoretical concept often attributed to the economist Olson (1971), is concerned with how and why individuals work together as a group when collective goals may be different from the interests of the individuals involved. Social norms are seen as only one factor among many that contribute to why individuals choose to cooperate towards achieving collective goals; others include levels of trust, past experiences, perceived benefits, local circumstances, culture, and ideology (de Marrais and Earle, 2017). Communicative approaches to collective action expand this even further in suggesting that collective action is embedded in time and space, and changes through different types of engagement and shifting motivations of actors (Flanagin, Stohl and Bimber, 2007).

Collective action theory has been utilised across disciplines, for example by political scientists to understand the motivations of rulers and state authorities in achieving social benefit for their populations (Levi, 2002), by archaeologists interested in the collective and cooperative aspects of human societies (Blanton and Fargher, 2016), and by anthropologists exploring the collective meanings attached to armed conflict and violence (Peregrine and Ember, 2016). For VAW intervention research, it is useful as a way of exploring the rationales of individual actors working towards a collective goal. Community mobilisation researchers have synthesized literature on collective action theory into conceptual tools for understanding why individuals participate in action against VAW when there might not be immediate benefits for themselves (Gram, Daruwalla and Osrin, 2019). More specifically, collective action theory pushes beyond an assessment of the rational choices made by individuals to explore the potential gains of group rationality that is ‘bounded’ by social norms (Ostrom, 2009). As such, it provides a useful conceptual tool for understanding *why* communities work to prevent VAW, and identifying additional structural or contextual barriers that may be preventing meaningful reductions in violence. It provides the ‘reasoning’ behind community-based mechanisms for violence prevention as part of our realist analysis, and contributes to understanding the interaction between mechanisms and context, described in more detail below.

## Methods

We present a realist analysis (Pawson and Tilley, 1997) of focus-group discussion (FGD) and in-depth interview (IDI) data, collected in four settings. Realist evaluation is a theory-driven approach, underpinned by Context-Mechanism-Outcome configurations (CMOs) that provide causal explanations of how change is triggered, for whom, and in what circumstances (Marchal *et al.,* 2012). Middle-range theories (MRTs) are developed to create an abstracted explanation of how change is achieved, which is transferrable across settings (Gilmore *et al.,* no date; Salter and Kothari, 2014). We employ these methods to help identify empirical examples of how communities act collectively to prevent VAW (outcomes) in particular settings (contexts), and the reasons why (mechanisms).

Although realist methodology is typically used for programme evaluation, we draw on its theory-driven principles and attention to programme strengths to guide this secondary data analysis (Kenten *et al.,* 2019; Kupeli *et al.,* 2019). We define context as local socio-cultural, economic and political factors that support or inhibit VAW prevention in the community. These could include strong community identities, inadequate housing, or policies targeted at communities. Mechanisms refer to explanations of how context influences stakeholder behaviour: the human reasoning underlying the behaviour change (Pawson and Tilley, 1997). For example, in a realist review of advocacy interventions for survivors of violence, mechanisms included survivors having trust in their advocate and feelings of self-efficacy (Rivas *et al.,* 2019). Lastly, outcomes refer to actions that communities take to prevent or respond to VAW, such as attending protests, providing emotional support to survivors, and reporting cases of violence to authorities (Singhal, Wang and Rogers, 2013; Wagman *et al.,* 2015). Our innovative use of the realist toolbox offers a structured method to explore common mechanisms of community-led VAW prevention across substantially different socio-cultural, economic and political contexts.

### Study settings

Data were collected from four separate research projects on VAW prevention in LMICs, led by coauthors (JM, DO, ND). ‘Community’ was defined similarly across projects: groups of people who are geographically and socially linked to a physical location (Rifkin, 2009). Data were collected in diverse locations including informal settlements in Mumbai, India, urban and rural locations in Afghanistan, the Peruvian Amazon, and urban communities in Kigali, Rwanda. The four socio-cultural, economic and political contexts are notably different. Recognising these contextual differences and considering their relevance to the outcomes we have identified is a key aim of our analysis.

Data from India were collected in 2019 as a sub-study of the SNEHA-TARA trial led by ND, KP, and DO, which is evaluating the impact of community mobilisation on VAW prevalence in informal settlements in Mumbai (Daruwalla *et al.,* 2019). While India is considered a lower-middle-income country (World Bank, no date), there are enormous socio-economic inequalities. In Mumbai, over 40% of available housing is located in informal settlements or slums (Central Bureau of Health Intelligence, 2019), which are characterised by substandard water and sanitation systems, environmental hazards, and inadequate housing (UN-Habitat, 2013). Women living in such settings are at increased risk of extreme poverty, malnutrition, poor mental health, and high levels of physical and emotional IPV (Daruwalla *et al.,* 2020).

Data from Afghanistan were collected in 2018-19 in a project led by AA, ND, CZ, JM, and DO to develop a package of care for survivors of VAW in South Asia (VAMHSA, 2019). A low-income country, Afghanistan’s persistent armed conflict has contributed to high levels of food insecurity and widespread trauma (Cooley, 2002). The conflict has also magnified deep-rooted gender norms that position women as the property of men and restrict their movement in public spaces through *mahram,* or guardianship by male relatives, first implemented by the Taliban (Ahmad and Anctil Avoine, 2018; Mannell *et al.,* 2020). Imprisonment and honour killings remain real risks for women who transgress social norms of marriage and household duties, including refusing to marry a man chosen by a father or guardian, having sex before marriage, or suspected adultery.

The Peruvian data were collected in 2017 during the Gender-based violence in the Amazon of Peru (GAP) Project led by JM, a partnership between University College London and local non-government organisation (NGO) DB Peru. Peru is an upper-middle-income country also characterised by stark inequalities, primarily between urban and rural settings. The Lower Napo River (LNR) basin, a rural area of the Amazon River, several hours by boat from Mazan (the closest semi-urban area), is populated by 25 small villages of roughly 5,000 people, many with ancestral ties to Peru’s indigenous communities. Most people in the area rely on subsistence agriculture and many women make and sell handicrafts at local markets. While the Peruvian government has established a network of 73 emergency centres that provide psychosocial, medical and legal support for women experiencing violence, these services are largely inaccessible for women in remote areas (Agüero, 2018).

Data from Rwanda were collected in 2013 during a study of community perceptions of gender-based violence (GBV) led by JM (Mannell and Jackson, 2014). Rwanda, classified as low-income, also has a history of armed conflict, with the 1994 genocide taking over 800,000 lives. As part of national efforts to rebuild the country and improve its international reputation, the Rwandan government takes a progressive stance towards women’s rights, implementing policies to prevent and punish perpetrators of GBV. These policies encourage survivors to report to community GBV committees, which are responsible for raising awareness, identifying and referring survivors to appropriate services, reporting perpetrators, conducting home visits, and reporting statistics on GBV cases to government authorities (Republic of Rwanda, 2011). Debate about GBV is also actively promoted by the police as part of government-mandated monthly community meetings (*umuganda*) and parents’ evenings (*umugoroba w’ ababyeyi*) (Mannell, Jackson and Umutoni, 2015; Mannell *et al.,* 2018). However, the women’s rights discourse used by the government has been critiqued for being at odds with the reality of women’s lives where widespread social norms continue to blame women for violence and deter survivors from seeking help (Umubyeyi *et al.,* 2016).

### Participant recruitment

In India, 13 women were recruited to participate in the HerStory project through the SNEHA-TARA trial. Community meetings were conducted with women involved in the trial and women who showed leadership qualities were invited to take up voluntary roles as *Sanginis* (female volunteers) and to take part in interviews. In Afghanistan, 90 men were purposefully selected to participate in FGDs through informal networks of an Afghan NGO committed to improving the health of women and children. Groups of men had diverse socio-economic characteristics, comprising university students, farmers, clinicians, and internally displaced individuals. In Peru, letters were distributed to 25 communities located along the LNR, inviting local community health workers and community leaders to an introductory GAP Project meeting. Eighty-one individuals attended these meetings. In Rwanda, two urban communities were selected based on research assistants’ local networks and predefined community (*umudugudu*) boundaries. Community chiefs recruited 23 men and women from their respective communities, and 12 members of a pre-established GBV Committee to participate in focus groups. A total of 232 participants was included in the analysis.

### Data collection procedures

In India, IDIs were conducted with women in Hindi by a local woman researcher from SNEHA using a predefined topic guide focusing on community responses to VAW. A vignette describing a situation where a woman was beaten publicly by her husband was used to elicit responses. In Afghanistan, FGDs were facilitated by a local woman researcher in Dari using a predefined topic guide focusing on understandings of VAW, conflict and mental health, and community responses to VAW. In Peru, community meetings were facilitated in Spanish by the GAP research team. Facilitators held a semi-structured group discussion on ‘What is gender-based violence?’, followed by an open discussion on ethical values for VAW prevention. In Rwanda, FGDs were facilitated by two local research assistants (male and female) in Kinyarwanda using a predefined topic guide, focusing on understandings of and responses to VAW and the role of GBV committees. All FGDs and IDIs were recorded and transcribed and translated into English. Ethical approval was received for all four studies and covers the secondary analysis of the datasets.

### Analysis

Analysis was an iterative collaboration between three researchers: HL, LB and JM. We individually read each transcript before discussing initial themes and a strategy for applying the CMO framework. We developed a theoretically-driven coding frame for deductive analysis based on whether the data pertained to context, mechanism or outcome. We then individually applied this to a specific country’s dataset using NVivo 12 (HL coded two datasets). Quotes were transferred to a shared Excel spreadsheet. Potential CMO configurations for each setting were discussed and refined by removing those not supported by evidence and merging those that overlapped conceptually.

Next, we synthesised these CMO configurations into MRTs. MRTs are theories at a higher level of abstraction, moving away from specific settings to provide a synthesis of observed mechanisms (Djellouli *et al.,* 2017). This was done by joining CMOs under common themes. In some cases, MRT support was provided through the absence of both mechanism and outcome (e.g., in Afghanistan there is little evidence of communities actively responding to VAW), and while this absence provides some evidence, we recognise this as a limitation of our results. The final MRTs were checked by returning to the data to ensure sufficient empirical support (and no alternative explanations). They were visualised as a conceptual diagram, reflecting generalisable mechanisms for community action to prevent VAW across the four settings. This framework was shared with co-authors who provided feedback to finalise it.

## Results

### Country-specific CMOs of VAW prevention and perpetuation

Table 1 presents example CMO configurations (column 2) that describe community actions to prevent, or perpetuate, VAW in India, Afghanistan, Peru and Rwanda. CMO configurations are presented under thematic categories (column 1), supported by illustrative quotations (column 3).

#### India

Families live in close proximity in urban informal settlements and community members can often see or hear when a woman is being physically or verbally abused. The density of communities in this setting appeared to influence how community members responded to VAW. Participants discussed how community members intervened to separate the couple, preventing the violence from escalating. However, community density sometimes had the inverse effect, deterring community members from intervening. Social norms defining violence as a private family matter result in community members worrying about the repercussions of getting involved in other families’ domestic matters. Participants discussed how this prevented community members from intervening altogether, while others provided informal support to couples and survivors in secret. There was also evidence of strict patriarchal norms positioning women as inferior to men in their families and communities. Participants recognised these norms as preventing women from reporting violence, creating an incentive for community members to speak up on their behalf.

#### Afghanistan

Little evidence of community action to address VAW surfaced in the men’s FGDs, with one exceptional example of a few men helping a woman obtain a divorce from her violent husband (divorce is often impossible for women in Afghanistan without the support of other more powerful men). Participants discussed the reasons why VAW is acceptable within limits, and while excessive physical violence was often discussed as unnecessary and wrong, the majority of men agreed it was widely accepted as a normal part of women’s lives. This is consistent with widespread social norms that position women as inferior to, and the property of, men. Participants themselves justified the use of VAW when it provided a means of maintaining women’s roles and inferior position vis-à-vis men. Here, participants reinforced the idea that VAW is acceptable, and explicitly stated that no help was provided for women experiencing violence.

#### Peru

In this context, an external NGO ran educational workshops with community health workers (CHWs) to raise awareness of VAW. During these workshops, participants developed knowledge around VAW and built confidence in discussing it, enabling them to share learnings with families and communities, raising community awareness of VAW and challenging related stigma and norms. During group discussions, participants talked about the NGO’s activities with CHWs, and explicitly called for dedicated VAW leadership within existing community leadership structures. Participants recognised the importance of community leaders responding to cases of VAW and acting as positive role models by setting a good example. The younger generation, a point of pride and central to community identity, was discussed as an incentive for tackling VAW. Participants recognised the negative impact VAW had on the lives of children, providing motivation to take action.

#### Rwanda

Rwanda has a strong domestic gender policy that specifically mandates the creation of community-based GBV committees tasked with responding to VAW cases. This context contributes to a strong sense of community identity and desire to tackle VAW for the collective good, discussed by participants as a trigger for community members intervening in cases of violence. Participants also said that support from local leaders on VAW-related matters facilitated community action to respond to violence. Specific examples were provided of instances where locally elected VAW committee members investigated and resolved cases of violence, and that monthly community meetings (*umuganda*) were used to discuss VAW-related matters. Participants trusted GBV committee members who were often well-respected elders. They felt that safe spaces for discussions of violence existed within their community, that they were able to report cases of violence and that there was a system in place to link families and survivors to higher levels of government support when needed.

### Middle-range theories of mechanisms supporting community actions to prevent VAW in LMICs

**Figure 1:**
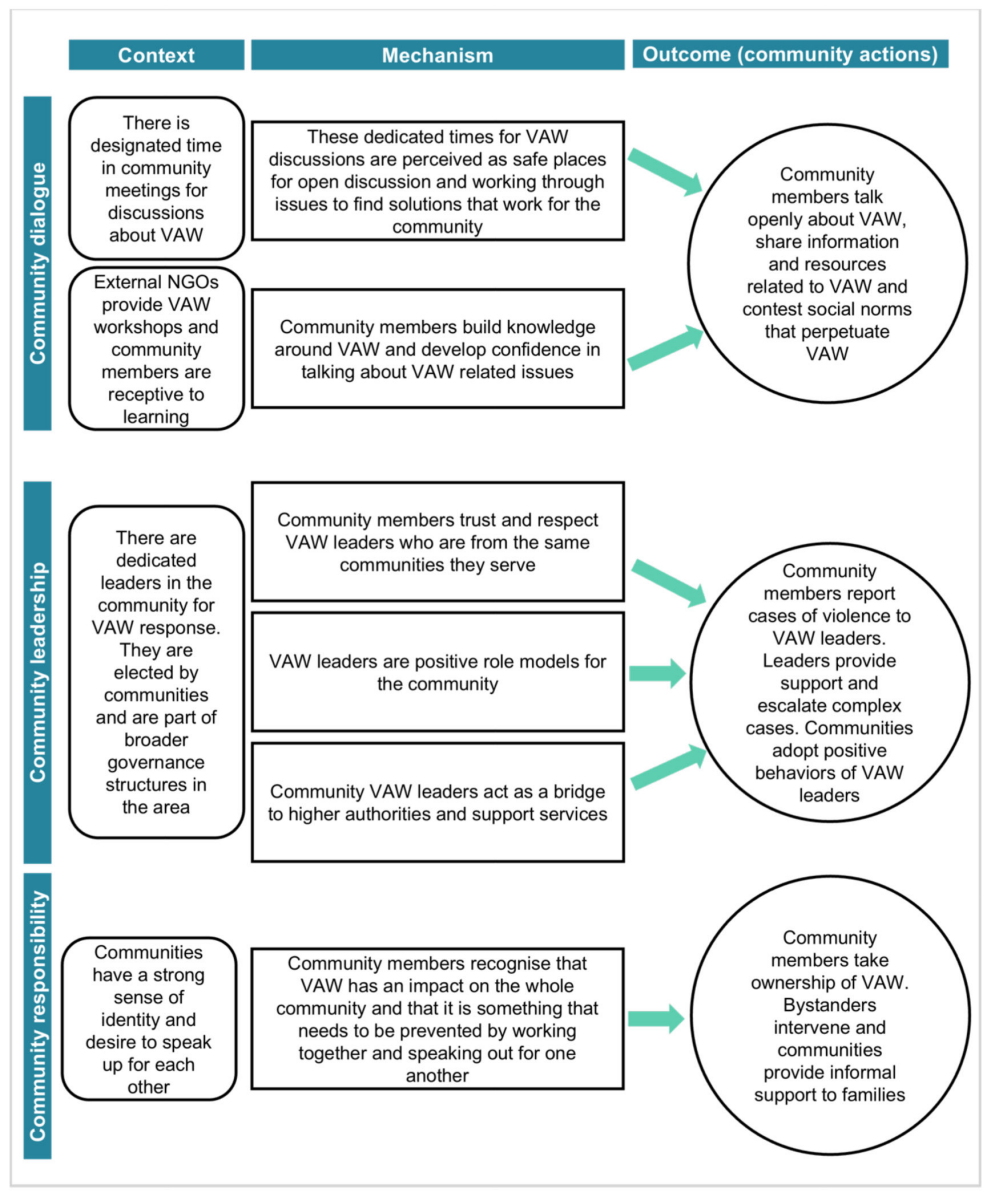
Conceptual framework of middle-range theories (MRTs) of community action to prevent violence against women (VAW) in low- and middle-income countries (LMICs).

The mechanisms supporting community actions to prevent VAW varied across settings. In this section, we present a synthesis of mechanisms supporting positive community responses to VAW as a conceptual framework based around three MRTs: community dialogue, community leadership and community responsibility (Figure 1). We also draw on specific examples where mechanisms failed to bring about community action because they were not triggered in the given context. Using examples from each dataset, we suggest how successful mechanisms might instigate community action to prevent VAW across LMICs.

#### Community dialogue

There were various examples of communities discussing VAW across the settings. However, in practice, the extent to which this dialogue supported community action to address VAW depended on the provision of resources for conversations to occur, socio-cultural norms that perceived VAW as a community issue, and broader social norms rejecting the use of VAW. For example, in Rwanda, public policies explicitly include provisions for community discussions about VAW, whereas in Afghanistan the context of a protracted war and instability has eroded social support networks.

The need for spaces for community conversations about VAW was recognised across all settings. Such spaces were perceived to provide the opportunity for sharing experiences of VAW, challenging the idea of violence as a private family matter. Several participants noted how overt discussions about violence occurred in their community:

*“It was only yesterday I had a debate with a man. He asked, what do you do here? I see you gathering women for meetings. I said, we are trying to create awareness among housewives. We speak with women who face violence inside their house. They come and tell us here.” [woman, IDI participant, India]*

Community dialogue was perceived as a means of facilitating communal accountability for VAW, fostering informal support for women, couples and families:

*“We talk to each other, about our family life, we talk and we share about our experience in the marital status because they are not similar, and we give advice to each other about how to behave.“ [women’s FGD participant, Rwanda]*

In this way, community dialogue provided a mechanism for reducing the stigma of VAW and instigating a responsibility shared between community members for preventing it. This may increase the reporting of cases of VAW, and ultimately increase support for violence survivors while deterring perpetrators.

The extent to which community dialogue was effective in instigating community actions to prevent VAW depended on whether violence was perceived as a community issue. Individuals across settings discussed instances where they didn’t respond to violence because they didn’t think it was their place to do so:

*“A couple mistreats their partner, then, due to the fact that he is my neighbour, I am going to keep quiet.” [man, mixed FGD participant, Peru]*

Similarly, community dialogue can also uphold VAW when discussions centre on justifying violence and reasons not to intervene. In these cases, social norms that condone acts of VAW are not challenged, cases are not reported, and survivors do not receive support, as suggested by participants across settings:

*“Generally people tend to say that the woman must’ve made some mistake, else why would a man beat his wife like that… What’s the big deal if a man slaps his wife.” [woman, IDI participant, India]*

*“Women do make mistakes. We have to be just. We have to take steps to keep our family together.” [men’s FGD participant, Afghanistan]*

As a result, the presence of community dialogue is insufficient to bring about community action to prevent VAW if the dialogue itself is informed by social norms condoning it.

#### Community leadership

Community leadership provides a mechanism for communities to take action towards addressing VAW. It sets a precedent for how communities should respond and creates local structures that support action. Community leadership to address VAW varied considerably across the settings, but evidence from Peru and Rwanda suggests this is an important mechanism. It was not possible to assess community leadership in India or Afghanistan because there was little evidence of response to VAW by leaders in these settings.

Data from Rwanda indicate that community leaders were respected, community members felt comfortable reporting violence to them and couples experiencing violence trusted and listened to them:

*“The first thing we do is alert the authority… this is a female who is in charge specifically for violence against women… So, the justice is not taken into the hands of the neighbour but the right person.” [men’s FGD participant, Rwanda]*

*“Young and old people respect her, and I was amazed at how they obeyed her and since then I haven’t heard of any conflicts in their home.” [women’s FGD participant, Rwanda]*

Similarly, trusting leaders was an important mechanism on the pathway to addressing VAW in data from Peru. In the context of a lack of trust in community authorities and no designated VAW leaders, communities lacked positive role models for violence prevention, resulting in a lack of motivation for community members themselves to take action:

*“He must be well mannered in the community, so that he is an example of security… so that you can set good examples… If the lieutenant behaves like this, how will the town walk? [woman, mixed FGD participant, Peru]*

In Rwanda, where community leadership was strong, leaders responded to and investigated cases of violence, provided counselling and continuous care for survivors and families, and escalated cases to higher authorities when needed. The presence of designated leaders with clearly defined roles in relation to VAW within the community sets a precedent and encourages communities to take action themselves.

#### Community responsibility

Across data from India, Peru and Rwanda, there were examples of communities recognising VAW as a problem requiring a community response. The extent to which this mechanism led to community action to address VAW was determined by the different contexts. In Rwanda and Peru, communities had a strong community identity and VAW was seen as an issue that needed preventing for the good of the community. In Rwanda, public policies govern community activities and make regular, formal community meetings compulsory for all community members. These meetings fostered a shared sense of accountability for community problems. Community members recognised the impacts of VAW for the whole community, providing a mechanism for community ownership of the problem and incentivising community members to take action against VAW:

*“... we all work together. We have frequent meetings to discuss the issues and we have one day for instance when there is a Umuganda, when we raise awareness of the GBV issues.” [women’s FGD participant, Rwanda]*

In data from Peru, where children were considered central to community identity, the need to prevent violence to protect children and future generations acted as a mechanism of community action:

*“We learn for ourselves; we learn for our children and for us to say to those who want to listen to us.” [man, mixed FGD participant, Peru]*

Conversely, the context that led to communities recognising the need to take action against VAW in India was different, triggering a different mechanism but resulting in a similar outcome: communities taking action. Here, restrictive patriarchal norms that participants recognised could render women unable to speak up about violence provided an incentive for communities to speak up on behalf of them:

*“In the atmosphere of patriarchy, respecting our elders, it is always women who suffer… when the fight breaks out… just by being a man, no one tells him off. But if a woman speaks up, she’s held at fault, because how dare she speak up against her father-in-law or husband.” [woman, IDI participant]*

There was a lack of recognition of VAW as a problem worth addressing in data from Afghanistan. As such, there was no context triggering a mechanism leading to community members intervening. For example, a participant spoke of VAW being normalised to the extent that violent acts were not considered as violence if they served to ‘correct’ women’s behaviour:

*“Not using violence has a limit. If a woman crosses her limit and doesn’t realize her position, then you have to be violent… If she does bad things then you have to use violence to teach her a lesson. This is not violence.” [men’s FGD participant]*

Examples of direct community action from Rwanda, Peru and India included community members turning up at the scene and trying to physically stop perpetrators, alongside reporting cases of violence to community leaders and other authorities:

*“If the husband is beating his wife, there is commotion. The neighbourhood can hear them. So, the neighbours start showing up.” [men’s FGD participant, Rwanda]*

*“So, if I see that someone is hitting the other person, well I am not silent, because by staying silent I am helping that person continue doing the same thing and it will not end” [woman, mixed FGD participant, Peru]*

When community members took responsibility for preventing violence in their own communities, collectively taking action against it by showing up and reporting, it sent the message that violence was not tolerated in the community and there would be consequences for perpetrators.

## Discussion

In developing realist methods for secondary qualitative data analysis, we have been able to assess the mechanisms that lead to community action to prevent VAW across LMICs. We have identified three overarching theories explaining how mechanisms of community action are triggered in different settings, based on CMO configurations from India, Afghanistan, Peru, and Rwanda. Opportunities for community members to discuss and build knowledge around VAW, trusted VAW leaders as positive role models, and community perceptions of VAW as a problem worthy of community intervention were important mechanisms on the pathway to community action to address VAW.

Our conceptual framework of collective community action (Figure 1) sheds light on how communities often work together in pursuit of a common goal and are not only a collection of individuals with independent motivations (Bandura, 2004; Campbell, 2021). When communities perceived VAW as a problem that needed addressing for the good of the whole community, they took ownership and shared responsibility for addressing it. This aligns with findings from a study of community readiness to tackle VAW in urban informal settlements in Mumbai (Gram *et al.,* 2020). It also has implications for evaluating VAW prevention interventions, in which less obvious forms of collective action such as shared awareness or discussions about VAW as a problem might not be assessed as intervention outcomes, despite making important contributions towards VAW prevention.

The differences in community action across settings highlight the importance of context as both a barrier and enabler to VAW prevention. The strongest evidence for community action comes from Rwanda and Peru, where contextual factors including policy and programmes targeting VAW prevention created an environment conducive to community action. In Afghanistan and India, where policies to prevent VAW were less evident at the community level, social norms about women’s position vis-à-vis men and violence as a private family issue inhibited community members from taking action against VAW, or even recognising it as a problem that required action. This points to a need for interventions that address structural and contextual risk factors for VAW (Mannell *et al.,* 2022), and attention to the national political and economic context as both an enabler and barrier to community members’ participation in collective action. In Rwanda, the government’s policy of mandating GBV committees in each village exists in a broader political context in which the government is monitoring local activities in pursuit of an agenda of gender equality, which has been widely criticised for its autocratic tendency (Gettleman, 2013). Examining the impact of this policy position, and the critiques leveraged against it, on the motivations for local community action in Rwanda, while beyond the scope of this analysis, would be an important avenue for further research.

Developing interventions without first assessing contextual factors and existing community activities can render interventions ineffective and even harmful (Craig *et al.,* 2018; Skivington *et al.,* 2021). Our findings support this, highlighting that in many cases, mechanisms for VAW prevention already exist within communities in LMICs, and offer an important starting point for new interventions. For example, in Rwanda, community leaders are central to the VAW response; their role is recognised by the community and they set the precedent for how communities should respond. An intervention that does not capitalise on this strong foundation of community leadership would be a missed opportunity for utilising existing mechanisms, and could also side-line leaders who would be essential to intervention success. Moreover, while social norms that firmly supported the use of VAW were pervasive in India and Afghanistan, there were still some mechanisms supportive of community action against VAW. In India, there was evidence in some cases that community members recognised violence as problematic, which led to actions such as providing informal support to a woman or couple. While these actions existed in the context of social norms positioning violence as a private family matter, they still suggest the presence of local mechanisms of change that could be harnessed in future interventions in this setting.

Our use of four different datasets has highlighted several strengths of using realist methods for cross-country comparisons. The different settings can be seen as representing different points along a pathway of change (Silva *et al.,* 2014), with each context leveraging some mechanisms and not others. Consistent with a realist approach to intervention evaluation, while all potential mechanisms exist, they may not be activated in particular settings depending on context (Djellouli *et al.,* 2017; Brand *et al.,* 2019). For example, mechanisms such as community dialogue about VAW have been activated in Rwanda where government policies already emphasise women’s rights, whereas the protracted war in Afghanistan and current Taliban leadership make it highly unlikely that Afghan communities will discuss VAW as a problem needing community involvement or mediation. The mechanism still exists, but is not activated. The capacity of a realist approach to develop middle-range theories from comparisons of how mechanisms operate within different contexts is one of the advantages of the method as an analytic strategy for systematising large qualitative datasets.

The conceptual framework arising from our analysis (Figure 1) makes a contribution to VAW research and practice by providing a useful model to assess existing mechanisms of change within communities when developing VAW prevention interventions at the community level (Fletcher *et al.*, 2016; Skivington *et al.,* 2021). While social norms are an explicit part of this, our conceptual framework extends beyond norms to include mechanisms such as trust and respect, safe spaces for discussion, recognition of the impact of VAW, and bridges to available resources outside the community. This strengths-based approach highlights communities as capable of positive change and places them at the heart of intervention development (Bryant *et al.,* 2021).

## Limitations

The study has some limitations. First, we synthesised datasets from four research projects with different aims and data collection methods. In India, data were collected with women, in Afghanistan with men, and in Rwanda and Peru with both men and women. Similarly, research participants had varying levels of engagement with VAW interventions, and therefore different exposure to messages about VAW prevention, which may have affected their responses to questions. Moreover, data were not collected with the aim of conducting realist evaluation and in some instances it was difficult to draw out CMO configurations because topic guides were not designed to ask questions about specific components of CMO analysis. This disconnect between data collection and analysis is a common challenge in secondary data analysis, and one which was overcome in this case by having a common outcome – community responses to violence against women – across the four studies. The realist approach also mitigated some of the challenges inherent in drawing comparisons across different settings and projects. The data collection methods and study aims are as much a part of the context as the settings where data were collected, and the realist approach provided a framework for including this in our analytical interpretation of the mechanisms.

## Conclusion

We present a conceptual framework of community mechanisms of VAW prevention across LMICs, based on empirical evidence from India, Afghanistan, Peru and Rwanda. This framework aligns with strengths-based perspectives, serving as a tool for assessing how communities might already be responding to VAW as part of intervention development research, so that their existing approaches are integrated into future interventions from the outset. Harnessing local mechanisms of change has great potential for ensuring relevance and long-term sustainability of interventions, centring community knowledge, and seeing these processes as facilitators rather than barriers to positive change towards community VAW prevention.

## Figures and Tables

**Table 1:** Example Context-Mechanism-Outcome configurations describing pathways to community action to address VAW in India, Afghanistan, Rwanda and Peru.

Theme	Context-Mechanism-Outcome (CMO) configuration	Supporting quotation(s)
India		
Support for couples may be provided in secret	**C:**Cultural norms consider VAW a private family matter **M:**Community members are motivated to help, but resist getting involved in other families’ problems **O:**Community members provide informal support to couples in secret	*Interviewer: “In your community, what tends to happen when someone hears that a woman is being abused physically or emotionally?” Participant: “I keep advising them in secret. I tell them what they should do, without letting my involvement come to the forefront.” [woman, IDI participant]*
Afghanistan		
Violence is necessary to maintain women’s roles	**C:**Social norms position women as the property and responsibility of men **M:**Violence is discussed as a legitimate means of maintaining women’s roles and inferior position vis-à-vis men **O:**Community members accept VAW as legitimate and blame women for transgressing social rules	*“I think if a woman makes a mistake then it is fine to beat her.” [men’s FGD participant]* *“A woman must be punished for her deeds” [men’s FGD participant]*
Peru		
VAW is a community problem	**C:**Children are central to community identity and communities are protective of their wellbeing **M:**Community members see the negative consequences of VAW for children **O:**Communities take ownership of preventing VAW for the future of their children	*“Violence is very bad for children, children are watching, […] children are already growing up in that environment, they are going to be violent too, one day when they have their wives, their children, they will do the same to them” [man, mixed FGD participant]*
Rwanda		
Community conversations about VAW	**C:**Formal community meetings take place regularly and the whole community are expected to attend **M:**They are perceived as safe spaces for discussing VAW and working together to find solutions **O:**Community members talk openly about VAW, raising awareness, breaking down stigma of talking about and reporting VAW, and allowing social norms that perpetuate VAW to be contested	*“The gatherings we have are important especially for women because the conversations can create the confidence that is needed for a woman to be able to communicate her problems.” [women’s FGD participant]* *“Sometimes we get together and talk and have a conversation as men about our community and the problems we face, we advise each other, and we see good results.” [men’s FGD participant]*
